# The Effects of Alcohol Intoxication on Neuronal Activation at Different Levels of Cognitive Load

**DOI:** 10.2174/1874440000802010065

**Published:** 2008-08-22

**Authors:** Hilde Gundersen, Renate Grüner, Karsten Specht, Kenneth Hugdahl

**Affiliations:** 1 Department of Biological and Medical Psychology, University of Bergen, Norway; 2 Department of Radiology, Haukeland University Hospital, Bergen, Norway; 3 Department of Physics and Technology, University of Bergen, Norway; 4 Department of Clinical Engineering, Haukeland University Hospital, Bergen, Norway; 5 Division of Psychiatry, Haukeland University Hospital, Bergen, Norway

**Keywords:** Alcohol intoxication, brain function, cognitive load, different blood alcohol concentrations

## Abstract

The aim of this study was to investigate how alcohol intoxication at two blood alcohol concentrations (BAC) affected neuronal activation during increasing levels of cognitive load. For this purpose we used functional magnetic resonance imaging (fMRI) together with a working memory n-back paradigm with three levels of difficulty. Twenty-five healthy male participants were scanned twice on two separate days. Participants in the control group (N=13) were scanned after drinking a soft-drink at both scanning sessions, while participants in the alcohol group (N=12) were scanned once after drinking an alcoholic beverage resulting in a BAC of 0.02%, and once after drinking an alcoholic beverage resulting in a BAC of 0.08%. A decrease in neuronal activation was seen in the dorsal anterior cingulate cortex (dACC) and in the cerebellum in the alcohol group at the BAC of 0.08% when the participants performed the most demanding task. The dACC is important in cognitive control, working memory, response inhibition, decision making and in error monitoring. The results have revealed that the effect of alcohol intoxication on brain activity is dependent on BAC and of cognitive load.

## INTRODUCTION

The effects of alcohol intoxication are in pervious behavioural studies shown to be most marked in situations involving abstract or complex contextual stimuli, when there are competition for processing resources, delayed responding, and shifting response contingencies [1, 2]. It thus seems that a common feature of tasks in which effects of alcohol intoxication are reliably observed, is when there is a need for cognitive control. This suggests that alcohol intoxication may have specific effects on brain processes involved in cognitive control, and particularly brain processes that require control of conflicting stimuli. Although previous behavioural studies have shown that effects of alcohol intoxication are dependent on cognitive load [1-3], the effects of alcohol intoxication on neuronal activation at different cognitive loads are not known. Cognitive load can be experimentally manipulated in a working memory task, with increasing number of items to be held in the active memory buffer. Maintaining of information in WM is thought to require attention [4], strategic processing such as rehearsal [5, 6] and active inhibition of simultaneously presented irrelevant information [7]. Weakening of attention, rehearsal and response inhibition are well-known aspects of impaired cognitive performance following alcohol intoxication [8-10]. A second argument for using a working memory n-back task was that the neuronal circuitry and cortical networks involved are fairly well outlined in fMRI and PET studies, revealing activation in dorsal anterior cingulate cortex (dACC), prefrontal-, and parietal corticies [11-14]. Working memory, thus, represents salient features of cognitive control and executive functioning [15, 16]. When considering critical brain regions that could be predicted to be affected by both cognitive load and alcohol intoxication, the dACC would be of special interest since previous studies have shown this region to be responsive to cognitive load [17] and alteration in neuronal activation is also shown following alcohol intoxication [18-20].

The main hypothesis in the present study was therefore that high cognitive load would modulate neuronal activation particularly in the dACC. Furthermore, it was expected that alcohol intoxication at the two blood alcohol concentration levels would have different neuronal and behavioural effects. To investigate effects of alcohol intoxication on behaviour and neuronal activation, a functional magnetic resonance imaging (fMRI) study was conducted with a working memory n-back task with increasing levels of difficulty, i.e. cognitive load (1-back, 2-back and 3-back). The n-back task involves concurrent storage and manipulation of information, which are the processes emphasized in contemporary theories of working memory [5]. Moreover, the BAC was varied in two levels, 0.02% and 0.08%, in order to investigate possible interactive effects between levels of cognitive load and levels of alcohol intoxication. There are no previous neuroimaging studies that have investigated effects at the BAC of 0.02% (see however Chamberlain and Solomon [21] for review of the few behavioural studies of BACs below 0.03%).

## MATERIALS AND METHODS

### Participants

Twenty-five right-handed, healthy male volunteers participated in the study. The participants were social drinkers, and none was dependent on nicotine, alcohol or other drugs. None of the participants had any known psychiatric or neurological disorder, and none was currently taking any kind of medication. The above information was obtained by the participants self-report on a questionnaire. Written consent was obtained from all participants, and the study was approved by the Regional Committee for Medical Research Ethics in Western Norway (REK-Vest) and by the Norwegian Data Inspectorate (NSD).

### Design

**Figures (1):**
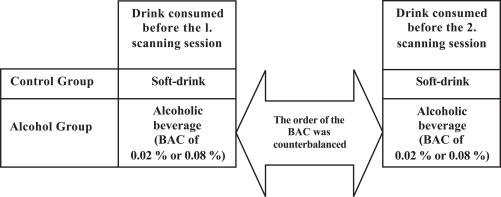
An outline of the design used. Participants in both groups were correctly informed about the content of the drink

Participants were randomly assigned either to the control group (N = 13, 28 ± 4 years and 78 ± 9 kg) or to the alcohol group (N = 12, 27 ± 4 years and 83 ± 6 kg). All participants were scanned twice on two separate days^1^. Participants in the control group were served a soft-drink before both scanning sessions, while participants in the alcohol group were served an alcoholic beverage resulting in a BAC of 0.02% or 0.08%. The order of the BAC obtained before each scanning session was counterbalanced across the participants (Fig. **1**).

### Procedure

All participants were instructed not to drink alcohol 24 hours before participating in the study. Moreover, they were instructed not to drink coffee, tea or coke four hours before participating since caffeine has neurochemical- and vasoactive properties that could otherwise have confounded the results. To avoid slow alcohol absorption, participants were also instructed not to eat a fatty meal two hours before participating.

Before being served the drink, the participants filled out informed consent and a questionnaire about handedness, age, bodyweight and educational level, about caffeine, nicotine and alcohol habits, and about drug abuse and medication. Before the first fMRI scanning session, all participants were tested on the forward and backward digit-span test from the Wechsler Adult Intelligence Scale (WAIS-R). This was done to control for individual differences in working memory span, which could be a confounding factor when solving the n-back task. To participate in the study, all participants had to achieve 100% correct on the 4-digits forward span test and at least 96% correct on the 3-digits backward span test, thus equalizing working memory span across participants. Detailed instructions about how to perform the working memory n-back task during the fMRI scanning session was also given to the participants before they were served the drinks. Task instruction was in addition displayed in the LCD-goggles that the participants wore during the scanning session, for 10 seconds at the beginning of each task.

Before the fMRI scanning session each participant was served a soft-drink or an alcoholic beverage, depending on group assignment (to avoid that expectation could confound the results, the participants knew the content of the drink). The drinks were individually tailored to the participant's bodyweight. The alcoholic beverage contained 60% pure ethanol diluted with tonic water, orange juice, cranberry juice and lemonade. Ethanol was replaced by a corresponding volume of tonic water in the soft-drink.

Blood samples were drawn from each participant for off-line analysis of glucose (Glu) (mmol/l) and haemoglobin (Hb) levels (g/dl). The blood samples were acquired before having the first drink, 30 minutes after having the last drink, and after the fMRI scanning session. This was done to control for possible group differences in blood Glu and Hb levels, which could affect the BOLD signal.

Breath alcohol concentration (BrAC) (mg/l) was determined before and after the fMRI scanning session, using the Evidenzer BrAC recording equipment (Nanopulse Inc, Sweden). BrAC measurements started 30 minutes after the drink was consumed, and continued every 5th minute until the same BrAC was obtained in two successive measurements, or until the level started to decrease. BrAC were then transformed to BAC.

### Stimuli and Experimental Design

**Figures (2):**
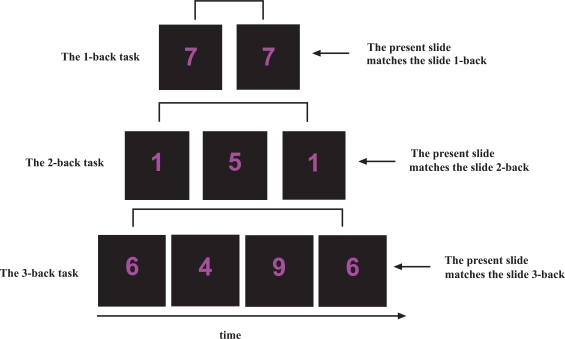
An outline of the n-back task used. In the 1-back task, participants were instructed to press a response button if the presented digit was the same as the digit presented 1 side back. In the 2-back task, participants were instructed to press a response button if the presented digit was the same as the digit presented 2-slides back. In the 3-back task, participants were instructed to press a response button if the presented digit was the same as the digit presented 3-slides back

The working memory n-back task used [12, 22] contained three separate runs, presented with an increase in cognitive load (1-back, 2-back and 3-back). In each run, single digits, from 1 to 9 were shown in the LCD goggles (NordicNeuroLab Inc., Norway) that the participants wore during the scanning session. Participants responded by pressing a response button on an electronic response grip (NordicNeuroLab Inc., Norway) held in their right hand whenever the presented digit was the same as the digit presented 1, 2 or 3 steps earlier in the sequence, depending on the instruction given (Fig. **2**). A block-design with six alternating ON and OFF blocks was used in each run, and each block lasted for 37.5 sec. Each ON-block consisted of presentation of 15 digits, giving a total of 90 digits per run. There were 3 target stimuli within each ON block, resulting in 20% target stimuli in each run. Each digit was presented for 300 ms, with a 2200 ms black screen between presentations. The OFF-blocks contained a black screen with the same duration as the ON-blocks, but with no task to perform.

The digit stimuli were programmed and presented with the E-Prime experiment control programming platform (Psychological Software Tools Inc., Great Britain). The timing of stimulus presentations was synchronized with the scanner image volume acquisition timing using a synchronization toolbox (NordicNeuroLab Inc., Norway). Response time (RT) and response accuracy (RA) were recorded and stored for subsequent off-line statistical analysis.

### fMRI Scanning

MR imaging was done on a 1.5 T Symphony scanner equipped with 30 mT/m quantum gradients (Siemens, Germany). Serial imaging with 185 BOLD sensitive EPI volumes were acquired during each run. Each EPI volume consisted of 28 axial slices acquired in ascending order, with 4 mm slice thickness and an interslice gap of 0.4 mm (FA / TR / TE / FOV / matrix=90^o^ / 2520 ms / 50 ms / 256 mm / 64 x 64 pixels) giving isotropic voxels of 4 mm^3^. Scanning of anatomy was done with a T1-weighted MPRage pulse sequence (FA / TR / TE / FOV / matrix = 15^o^/ 1910 ms / 3.93 ms / 256 mm/ 256 x 256 pixels) giving isotropic voxels of 1 mm^3^.

### Data Analysis

#### fMRI Data

Image processing and data analysis were performed using the Statistical Parametric Mapping (SPM2) software package (Wellcome Department of Cognitive Neurology, London, UK) and MATLAB6.5 (Mathworks Inc., USA). The first five volumes for each run were excluded before pre-processing of the data, to get a steady state signal. To correct for head movements, a series of data pre-processing steps were done in which the EPI-images were realigned intra-individually on a voxel-by-voxel basis to the first image in the second run.**The realigned EPI-images were then normalised (3 mm^3^) into the MNI standardized stereotaxic space (template provided by the Montreal Neurological Institute, Canada), and smoothed (Gaussian, FWHM 8 mm).**The**EPI-images were high-pass filtered (128 sec) to remove artifacts due to cardio-respiratory and other cyclical influences.

For the single-subject analysis, a fixed-effects model was used where the expected hemodynamic response was modeled with a canonical hemodynamic response function (hrf) according to the block design to create covariates in the General Linear Model. For the group-analyses, a random-effect model based on the contrast images from the single-subject analyses was used.

Two-sample t-tests were performed to compare the control group with the alcohol group at both BACs. In order to test the prediction of reduced processing capacity in brain processes that underlie stimulus conflict and cognitive control following alcohol intoxication, the results were explored with an uncorrected significant level of p < 0.001, and with an extent threshold of 10 voxels. In all analyses, the OFF condition (black screen) was used as the control condition.

The reported voxel coordinates were transformed from MNI space to Talairach space, and validated against the Talairach and Tournoux atlas [23]. The resulting set of t-values constituted the statistical parametric map (SPM2).

Based on findings from previous neuroimaging studies on alcohol intoxication [18-20], differential neuronal activation was expected in the dACC. To statistically compare neuronal activation between the control group and the BAC of 0.02% and 0.08%, a volume of interest (VOI) analyses was performed. The VOI for the dACC was defined from a significant cluster obtained in the control group when solving the 3-back task and compared to the BAC of 0.02% and 0.08%. To calculate if there were significant differences in the dACC between the groups in the 1-back, 2-back and 3-back tasks, the Tukey’s HSD post-hoc test was used. To control that between-group differences in the dACC were not caused by a general difference in base-line levels, caused by a general effect of alcohol onto the BOLD signal, the same analysis was conducted for a VOI in the occipital region. No paradigm related neuronal activation was expected for this latter region.

#### Behavioral Data and Blood Samples

One-way ANOVA and Tukey’s HSD post-hoc test were used to analyze group differences in RA and RT, and in blood Glu and Hb levels (Statistica 7.0, StatSoft Inc. USA).

## RESULTS

### Comparing the Control and the Alcohol Group at the BAC of 0.08%

When subtracting neuronal activation obtained in the control group from neuronal activation in the alcohol group, there remains no activation either when performing the 3-back task or the 1-back task. When performing the 2-back task, there was remaining neuronal activation in small clusters the right middle occipital gyrus (MOG) and in the left cuneus in the alcohol group (Table **1**).

**Figures (3):**
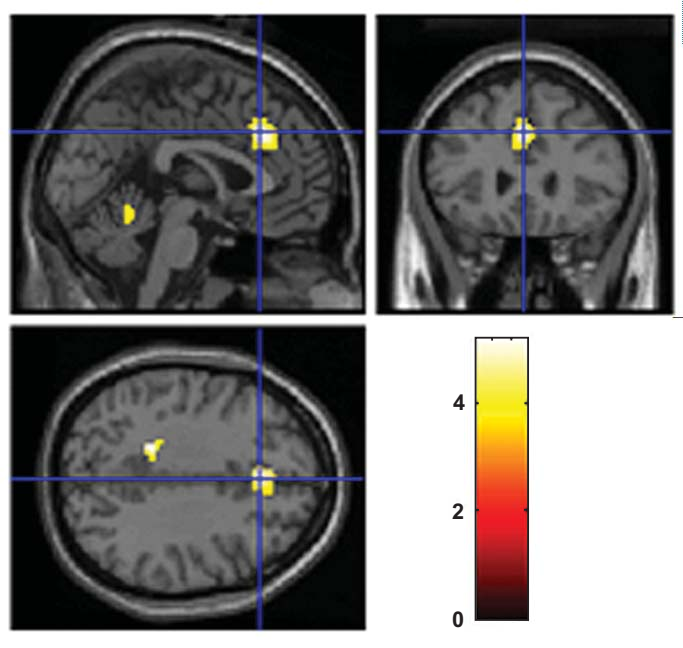
Remained neuronal activation in the control group compared with the alcohol group at BAC of 0.08% when solving the 3-back task. A two sample t-test with an uncorrected significance level of p< 0.001 and with an extent threshold of 10 voxels was used. The color coding indicates the t-value scores

When subtracting neuronal activation obtained in the alcohol group from neuronal activation in the control group, there was remaining activation bilaterally in the dACC and in cerebellum when performing the 3-back task (Fig. **3**). There remained no neuronal activation in the control group either when performing the 1-back task or the 2-back task (Table **1**).

### Comparing the Control Group and the Alcohol Group at the BAC of 0.02%

When subtracting neuronal activation obtained in the control group from neuronal activation in the alcohol group, there was remaining activation in the alcohol group in a small cluster in the left caudate nucleus when performing the 3-back task. When performing the 2-back task, there was remaining neuronal activation in the right caudate nucleus, in the left MOG and superior occipital gyrus (SOG) (Table **2**).

When subtracting neuronal activation obtained in the alcohol group from neuronal activation in the control group, there remained no activation (Table **2**).

### VOI Analysis

**Figures (4):**
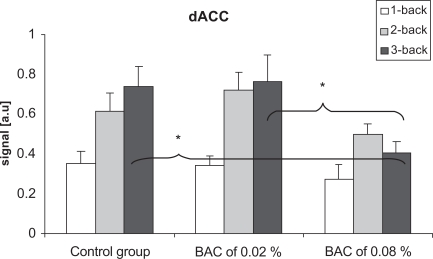
Differences in the signal in the dACC in the control group and at BACs of 0.02% and 0.08%. There were no significant differences between the groups when performing the 1-back and the 2-back task. When performing the 3-back task, there were significant decrease in the signal at the BAC of 0.08% compared to the two other groups. Vertical bars represent standard error of the means.*p < 0.05.

There were significant differences in neuronal activation in dACC between the control group and the alcohol group at the BAC of 0.08% (p = 0.047), and between the BAC of 0.02% and 0.08% (p = 0.036) when performing the 3-back task. Furthermore, there was no significant difference between the control group and the alcohol group at the BAC of 0.02%. There was no significant difference in neuronal activation in dACC between the groups when performing the 1-back and 2-back tasks (Fig. 4). Within-group analyzes showed that there was a significant increase in neuronal activation in the dACC between the 1-back task and the 3-back task (p= 0.004) in the control group. Moreover, there were significant increases in neuronal activation between the 1-back task and the 2-back task (p=0.001), and between the 1-back task and the 3-back task (p=0.001) at a BAC of 0.02%. In the inferior occipital cortex, there was no significant difference between the groups, neither with respect to BAC level nor cognitive load.

### Behavioural Data

There were no significant differences between the control group and the alcohol groups, neither for RA nor for RT. However, there was a trend towards more errors in the alcohol group at the highest BAC level. The means for RT and RA, split for groups, cognitive loads and BACs are seen in Tables **3** and **4**.

### Blood Samples

There were no significant differences between the groups, neither in blood Glu level nor in blood Hb level. The blood Glu level increased for all groups, independent of whether the participants were drinking the soft-drink or the alcoholic beverages, and decreased back to base-line after the fMRI scanning session (Table **5**). There were no changes in blood Hb level between the groups in the three measurements (Table **6**).

## DISCUSSION

Alcohol intoxication at the BAC of 0.08% caused decrease in neuronal activation, particularly in the dACC and in the cerebellum, although the decrease was only seen during the most demanding 3-back task. There were no regions with decreased neuronal activation following alcohol intoxication at the BAC of 0.02%. Increased neuronal activation following alcohol intoxication was seen in occipital regions when performing the 2-back task at the BAC of 0.02% and 0.08%, and in the caudate nucleus when performing the 2- and 3-back task at the BAC of 0.02%.

Neuronal activation in dACC was, as expected, increasing from the 1-back task to the 3-back task in the control group. The increase in neuronal activation was also significant at the BAC of 0.02%. In contrast, there was no significant increase in neuronal activation in dACC from the 1-back task to the 3-back task at the BAC of 0.08%. Although previous neuroimaging studies also have shown decrease in neuronal activation in the dACC following alcohol intoxication [18-20], the novel finding in the present study is that the decrease is dependent on cognitive load. Furthermore, the current results also showed that the decrease only was seen at the BAC of 0.08%. That alcohol intoxication affects neuronal activation in the dACC in a dose-dependent way has previously been reported by Calhoun *et al*. (2004a, b) who studied BACs of 0.04% and 0.08%, and by Ridderinkhof *et al*. (2002) who studied BACs of 0.04% and 0.1%. The novel finding in the present study is that a BAC of 0.02% not effects neuronal activation.

Interestingly, the results are indicating that alcohol is mainly affecting the execution of the task, as reflected in the decrease of the dACC activation, while the remaining prefrontal and parietal areas are not affected. This, in addition, is highlighting the selective effect of alcohol on the executive system. The activation in the alcohol group seen in the occipital and cuneus areas may be linked to the attention aspects of the tasks, since working memory tasks also depend on attention monitoring. The caudate nucleus has also been shown to be involved in learning and memory functions [24], in addition to its role in control of voluntary movements.

The results are indicating that the anterior cingulate cortex (ACC) may be particularly vulnerable to the effects of alcohol intoxication. Decrease in neuronal activation is not only seen following alcohol intoxication in social drinkers, but also in abstinent alcohol dependent individuals [25]. Moreover, alteration in brain metabolites in dACC has been observed in young alcohol dependent individuals. Interestingly, the latter finding has been correlated with altered short-term memory functions [26]. Decrease in glucose metabolism has also been shown in the cingulate cortex [27], and shown to correlate with neuropsychological performance. Thus, the decrease in neuronal activation observed in dACC following alcohol intoxication in social drinkers in the present study, may contribute to the understanding of the observed impairment in response inhibition, error monitoring, decision making and working memory seen in social drinkers and also in alcohol dependent individuals.

The present results also showed a decrease in neuronal activation in the medial cerebellum at BAC of 0.08% in the most demanding 3-back task. The cerebellum is known to show enhanced activation with increasing memory load [28, 29], and there is mounting evidence that the cerebellum participates in higher-order cognitive tasks such as executive processing, working-memory, verbal fluency and planning [30].

Participants in the control group showed neuronal activation in regions known to be involved in working memory[11-14], which validates the paradigm used. Moreover, there was, as expected, an increase in neuronal activation (both in intensity and in extent) from the 1-back task to the 3-back task.

A potential confounding factor in all fMRI-studies on alcohol intoxication is that alcohol in itself has vasoactive properties. Increase in cerebral blood flow (CBF) in specific brain regions following alcohol intoxication was shown in a previous perfusion MRI study in our laboratory using the same procedures and a BAC of 0.08% (Gundersen *et al*., submitted). Such regional increases in CBF could be expected to elevate the baseline signal (the “OFF condition”) in an fMRI BOLD experiment due to the presence of an increased level of oxygenated blood. However, it is not clear if this would eventually affect the BOLD signal, as the BOLD signal primarily reflects task-related and relative hemodynamic changes. In the current study, both blood Glu- and Hb levels were the same for all groups, so a difference in these parameters could also not explain the differences in the results. 

As a side-effect of the current results it could be argued that new, knowledge of how alcohol intoxication affects neuronal activation at different cognitive loads may also contribute to the ever ongoing discussion of accepted BAC levels in society at large, e.g. for traffic legislation. Driving performance involves complex cognition, including online working memory, and the driver need to react swift and flexible to the complex cues present in the modern traffic pattern [31]. In addition to being involved in working memory, dACC is involved in cognitive control, response inhibition, in decision making and in error monitoring [32], which all are properties required for adequate driving performance.

It would be interesting in future studies to investigate how alcohol intoxication affects other cognitive processes than working memory, especially when using tasks with different cognitive load in e.g. attention or executive tasks. In addition it would be interesting to investigate how other BAC levels affect neuronal activation, as well as investigating the effects of sex by also including females.

## Figures and Tables

**Table:** Table 1. Neuronal Activation when Comparing the Control and Alcohol Group at BAC of 0.08%

The 3-back task; group differences								
Remaining Neuronal Activation in the Control Group								
Statistical Values			Coordinates			Anatomical Location		
Cluster Size	*t-* value	*p-* value	*x*	*y*	*z*	Hemisphere	Structure	Brodmann Area
99	5.21	0.001	0	28	32	Left	dACC	32
	3.66	0.001	9	20	40	Right	dACC	32
22	5.06	0.001	-18	-36	35	Left	MCC	31
20	4.44	0.001	6	-53	-10	Right	Cerebellum	
**Remaining Neuronal Activation in the Alcohol Group**								
*No significant activation*								
**The 2-back task; group differences**								
**Remaining Neuronal Activation in the Control Group**								
*No significant activation*								
**Remaining Neuronal Activation in the Alcohol Group**								
**Statistical Values**			**Coordinates**			**Anatomical Location**		
**Cluster Size**	***t-* value**	***p-* value**	*x*	*y*	*z*	**Hemisphere**	**Structure**	**Brodmann Area**
13	4.53	0.001	45	-82	2	Right	MOG	19
	3.82	0.001	42	-84	10	Right	MOG	19
12	3.87	0.001	12	-95	16	Right	MOG	18
15	3.76	0.001	-3	-95	24	Left	Cuneus	19
**The 1-back task; group differences**								
**Remaining Neuronal Activation in the Control Group**								
*No significant activation*								
**Remaining Neuronal Activation in the Alcohol Group**								
*No significant activation*								

dACC = dorsal anterior cingulate cortex; MCC = midcingulate cortex; MOG = middle occipital gyrus.

**Table:** Table 2. Neuronal Activation when Comparing the Control and Alcohol Group at BAC of 0.02%

The 3-back task; group differences								
Remaining Neuronal Activation in the Control Group								
*No significant activation*								
Remaining Neuronal Activation in the Alcohol Group								
Statistical Values			Coordinates			Anatomical Location		
Cluster Size	*t*-value	*p-* value	*x*	*y*	*z*	Hemisphere	Structure	Brodmann Area
10	4.18	0.001	-6	17	-1	Left	Caudate	
**The 2-back task; group differences**								
**Remaining Neuronal Activation in the Control Group**								
*No significant activation*								
**Remaining Neuronal Activation in the Alcohol Group**								
**Statistical Values**			**Coordinates**			**Anatomical Location**		
**Cluster Size**	***t*- value**	***p-* value**	*x*	*y*	*z*	**Hemisphere**	**Structure**	**Brodmann Area**
57	4.33	0.001	6	15	-1	Right	Caudate	
33	4.13	0.001	-27	-77	31	Left	MOG	19
	4.04	0.001	-24	-71	42	Left	SOG	7
**The 1-back task; group differences**								
**Remaining Neuronal Activation in the Control Group**								
*No significant activation*								
**Remaining Neuronal Activation in the Alcohol Group**								
*No significant activation*								

MOG = middle occipital gyrus; SOG = superior occipital gyrus

**Table:** Table 3. Mean RA (%) in the 1-Back, 2-Back and 3-Back Task

	1-back	2-back	3-back
**Control group **	98 ±3	97 ±7	84 ±15
**BAC of 0.02 %**	95 ±6	95 ±6	75 ±19
**BAC of 0.08 %**	95 ±9	90 ±12	70 ±19

**Table:** Table 4. Mean RT (ms) in the 1-Back-, 2-Back- and 3-Back Task

	1-back	2-back	3-back
**Control group **	531 ±141	557 ±107	653 ±184
**BAC of 0.02 %**	550 ±109	538 ±109	672 ±174
**BAC of 0.08 %**	552 ±71	595 ±135	686 ±153

**Table:** Table 5. Mean Blood Glu Levels (mmol/l) in the Different Groups

	Before Consumption of the Drink	After Consumption of the Drink	After the fMRI Scanning Session
**Control group**	5.3 ± 0.7	7.4 ± 1.5	5.5 ± 0.9
**BAC of 0.02 %**	4.9 ± 0.8	7.1 ± 1.8	5.2 ± 0.8
**BAC of 0.08 %**	5.0 ± 0.6	7.4 ± 1.1	4.9 ± 1.0

**Table:** Table 6. Mean BLOOD Hb Levels (g/dl) in the Different Groups

	Before Consumption of the drink	After Consumption of the Drink	After the fMRI Scanning Session
**Control group**	15.2 ± 1.1	15.1 ± 1.0	15.2 ± 0.9
**BAC of 0.02 %**	15.3 ± 1.2	15.6 ± 0.8	15.8 ± 1.1
**BAC of 0.08 %**	15.6 ± 0.8	15.9 ± 0.7	16.1 ± 0.9

## Footnotes

To control for possible learning effects caused by repeating the working memory task twice (at BACs of 0.02% and 0.08% for participants in the alcohol group), participants in the control group was also scanned twice. The control group which was used in the analysis consisted thus of neuroimaging data obtained in the first scanning session for seven of the participants and of neuroimaging data from the second scanning session for six of the participants.
